# Limited accuracy of transtibial aiming for anatomical femoral tunnel positioning in ACL reconstruction

**DOI:** 10.1051/sicotj/2025002

**Published:** 2025-02-10

**Authors:** Dimitrios Mastrokalos, Anastasios G. Roustemis, Dimitrios Koulalis

**Affiliations:** 1 ATOS Klinik Heidelberg, Internationales Zentrum für Orthopädie Bismarckstraße 9-15 69115 Heidelberg Germany; 2 General University Hospital ATTIKO, 1st Surgical Orthopaedic Department of N.K.U.A. Rimini 1 Chaidari 12462 Greece

**Keywords:** Knee arthroscopy, Anterior cruciate ligament, Single-bundle ACL technique, ACL femoral tunnel positioning, Transtibial femoral tunnel

## Abstract

*Background*: Anterior cruciate ligament (ACL) rupture is a common knee injury, and with advancements in knee arthroscopy, ACL reconstruction has become common. Techniques like single-double bundle and femoral tunnel drilling via transtibial or anteromedial portal approaches are available. This study evaluates the accuracy of femoral tunnel placement via these approaches in single-bundle ACL reconstruction. *Materials and methods*: Forty-three ACL reconstructions using hamstring grafts were analyzed. Initially, femoral tunnels were drilled via the anteromedial portal from 09:30 to 10:00 (14:00 to 14:30 for left knees). Tibial tunnels (mean anteroposterior angle: 63.5°, sagittal: 64.2°) were then created with the same diameter, accompanied by radiological documentation. A femoral aiming device was used to place a K-wire at the center of the femoral tunnel, recorded photographically. Tunnel diameters included 7 mm (20 cases), 7.5 mm (11 cases), 8 mm (7 cases), 8.5 mm (3 cases), and 9 mm (1 case). Two observers evaluated all radiological and photographic data, focusing on the deviation of the transtibial K-wire from the femoral tunnel center. *Results*: Of 38 evaluated cases, the transtibial K-wire was within the femoral tunnel in 11 cases (28.9%) – 7 cases with 7 mm, 2 cases each with 7.5 mm and 8 mm diameters. In 23 cases (60.5%), the K-wire was at the perimeter or outside the femoral tunnel – 11 cases with 7 mm, 8 with 7.5 mm, 4 with 8 mm, 3 with 8.5 mm, and 1 with 9 mm diameters. *Conclusion*: Transtibial aiming for anatomical femoral tunnel positioning is challenging. No significant correlation was found between the transtibial deviation and the tibial tunnel diameter.

## Abbreviations

ACL: Anterior Cruciate Ligament
LIT: Lateral Instability of the Tibia
K-wire: Kirschner Wire
PCL: Posterior Cruciate Ligament

## Introduction

The knee joint’s kinematics and mechanics are inherently complex, making their surgical replication challenging [1, 2]. Traumatic events that damage the menisci and ligaments often accelerate joint degeneration [2, 3]. Among these injuries, anterior cruciate ligament (ACL) rupture is particularly common, prompting ongoing efforts to develop effective repair techniques [3–5]. Various approaches for graft placement have been described, including the transtibial method and independent femoral tunnel techniques [4–6]. Additionally, the choice of graft type and the use of single- or double-bundle reconstruction remain key considerations in ACL repair.

The main techniques for femoral tunnel creation are the transtibial and anteromedial portal approaches [7]. The anteromedial portal technique offers flexibility and precision for anatomically and functionally optimal femoral tunnel placement, making it ideal for anatomic and all-inside ACL techniques, whereas achieving similar precision with the transtibial technique is more challenging [8, 9].

The ACL consists of two bundles: the anteromedial bundle, which prevents anterior tibial translation in flexion, and the posterolateral bundle, which resists anterior translation in extension and controls rotational instability [10]. ACL reconstruction aims to restore both anterior stability and rotational control [10]. Recent trends emphasize reconstructing both ACL bundles to restore both linear and rotational stability [3]. However, single-bundle reconstruction has shown limitations in restoring rotational stability [11], and studies have noted its effects on tibial rotation during dynamic activities such as pivoting and descending stairs [12]. Because of the challenges of double-bundle reconstruction due to extensive bone trauma, attempts have been made to achieve similar stabilizing benefits through single-bundle techniques by positioning the femoral tunnel more posteriorly, mimicking the posterolateral bundle [7].

Although ACL reconstruction has been performed for many years using both transtibial and independent femoral tunnel techniques, few studies have directly measured the intraoperative accuracy of these methods [3, 7, 8, 13]. The primary aim of this study was to evaluate the accuracy of femoral tunnel placement using the transtibial approach compared to the anteromedial portal approach in single-bundle ACL reconstruction, with a focus on assessing the reliability of the measurements obtained through radiological analysis.

## Materials and methods

### Materials

This study included 43 patients (44 knees) who underwent ACL reconstruction using hamstring grafts performed by the same surgeon (H.H.P.) during 2004 and 2005. The cohort consisted of 24 men and 19 women, with a mean age of 34.8 years (range: 15–55 years). All patients were evaluated arthroscopically, and any concomitant lesions such as meniscal tears or chondral defects were treated prior to ACL reconstruction. The hamstring grafts were harvested and prepared as quadrupled grafts for single-bundle ACL reconstruction [14]. Radiological and photographic documentation was performed throughout the procedure to evaluate tunnel placement accuracy.

### Methods

#### Intraoperative stage

**Figure 1:**
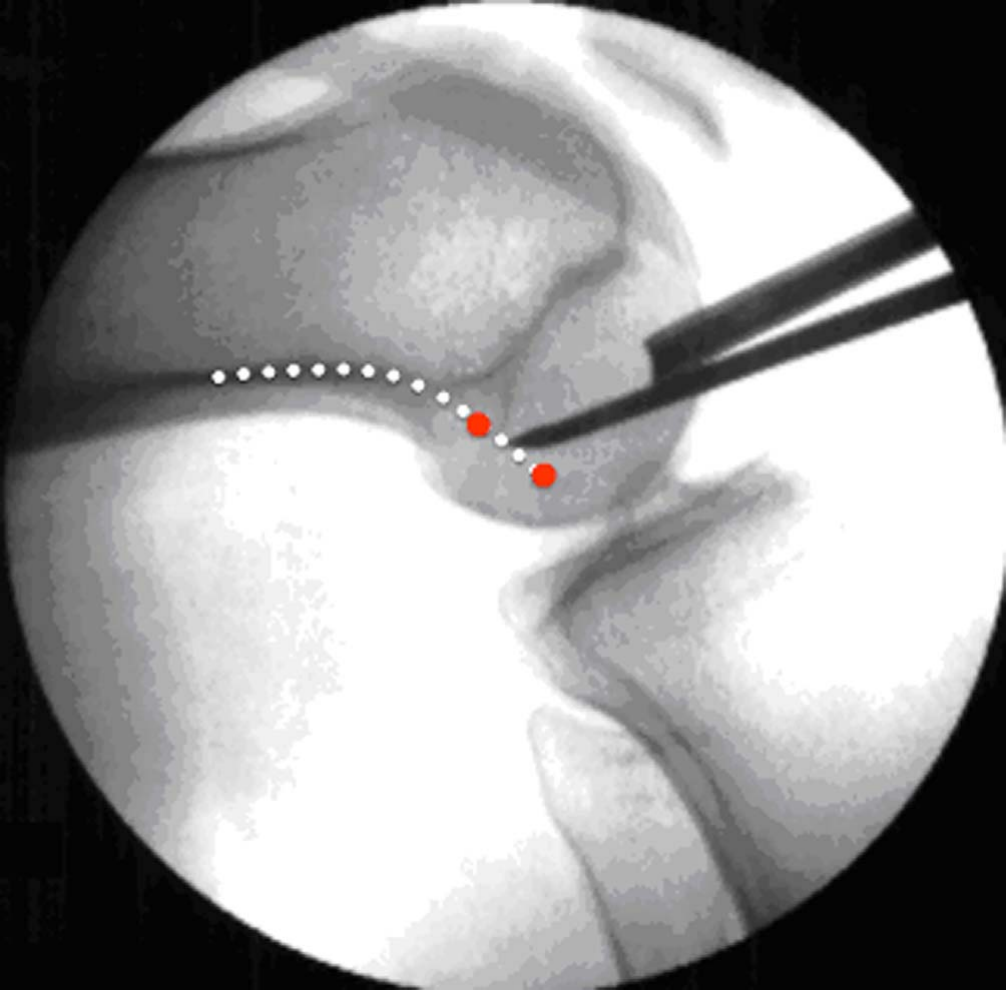
Knee flexion to 120° with subsequent drilling with the K-wire from the proximal femoral cortex, replaced by an elastic Nitinol wire.

After removal of the ACL remnants, leaving small proximal and distal stumps where possible, the femoral tunnel was prepared. Using a 5-mm offset femoral aiming guide through the anteromedial portal, the femoral ACL insertion was targeted at 90° of knee flexion, aiming for 09:30 (or 14:30 for left knees). Fluoroscopy confirmed the K-wire position, ensuring it was slightly inferior to the most superoposterior quadrant (greater than 25%) and approximately a quarter of the notch height (greater than 28.5%) from the roof [9]. The femoral tunnel was then drilled at 120° of knee flexion using a drill bit matching the graft diameter (Figure 1).

**Figure 2:**
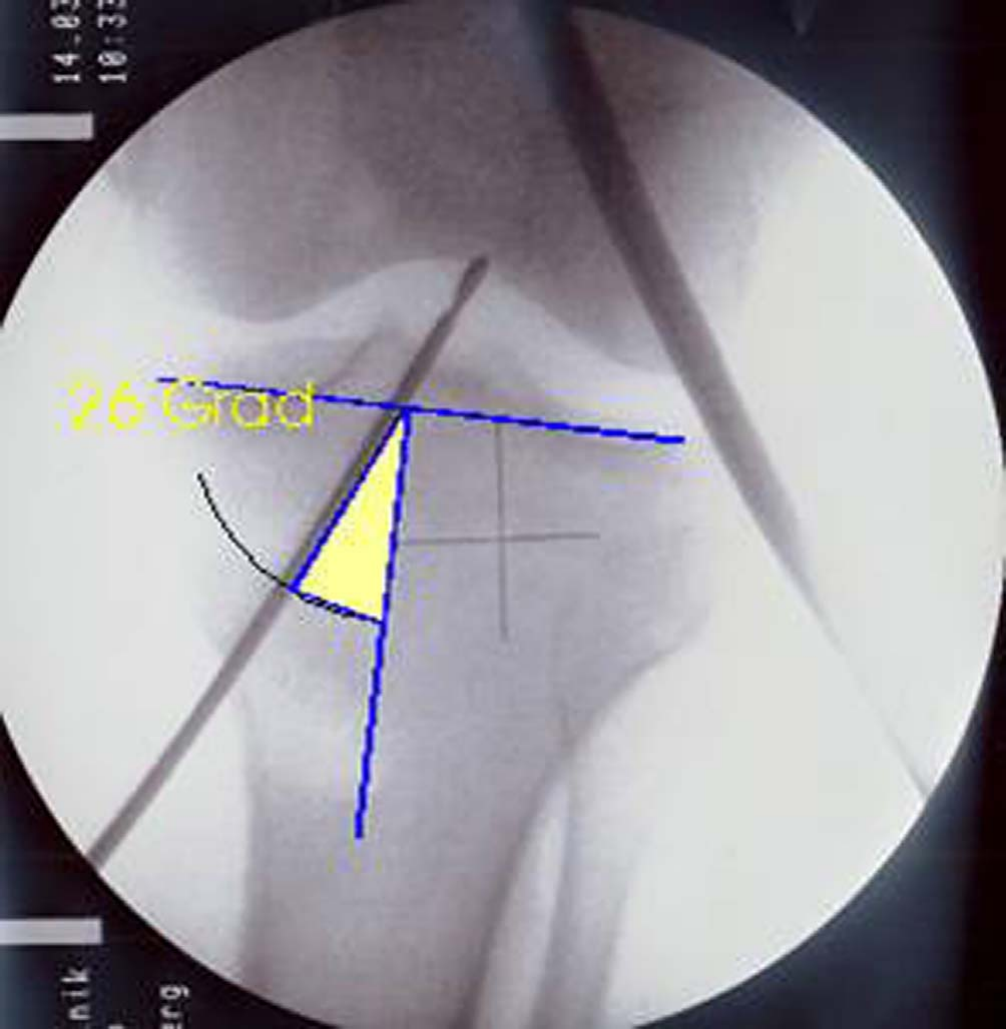
Tibial tunnel drilled at 50° with guide sleeve positioned 1 cm above the pes anserinus and 1.5–2 cm medial to the tibial tubercle for optimal graft length.

**Figure 3:**
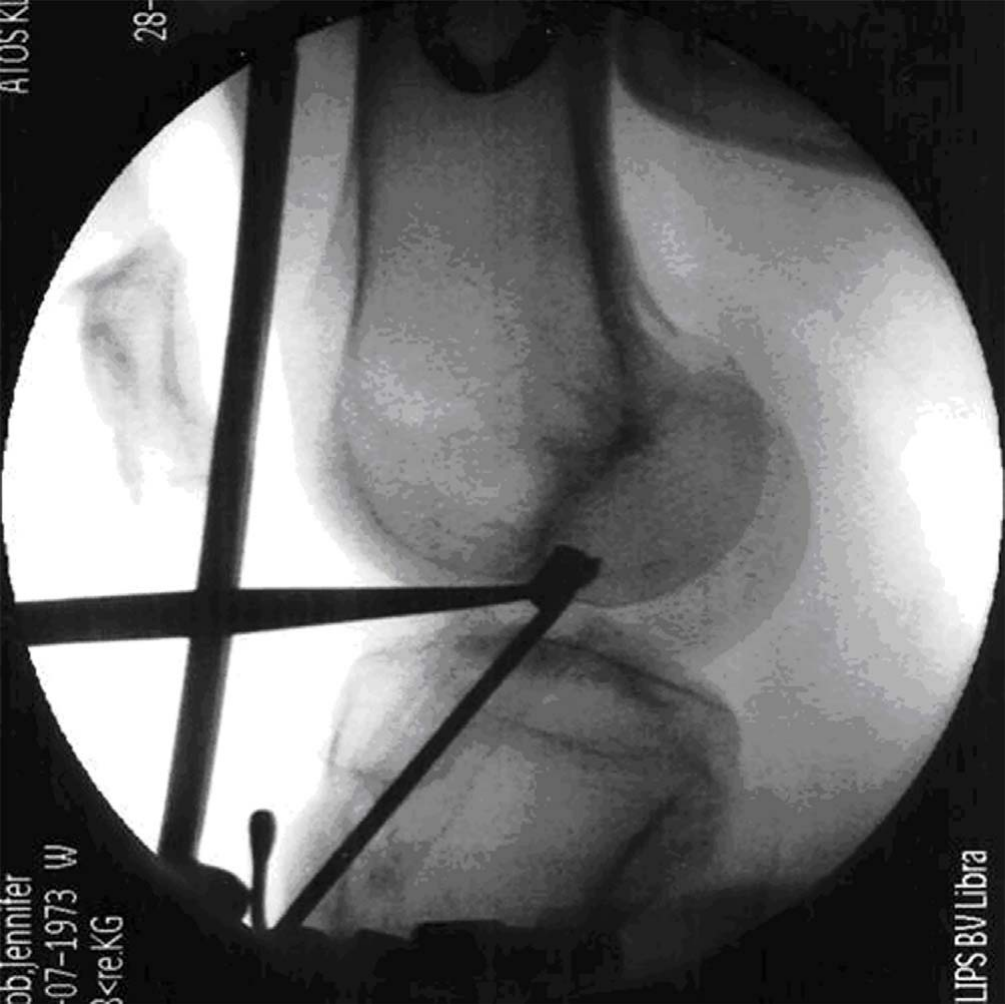
Tibial tunnel angle set to 65° in both coronal and sagittal planes, with K-wire aimed lateral to the medial tibial spine, aligned with the posterior rim of the anterior horn of the lateral meniscus.

**Figure 4:**
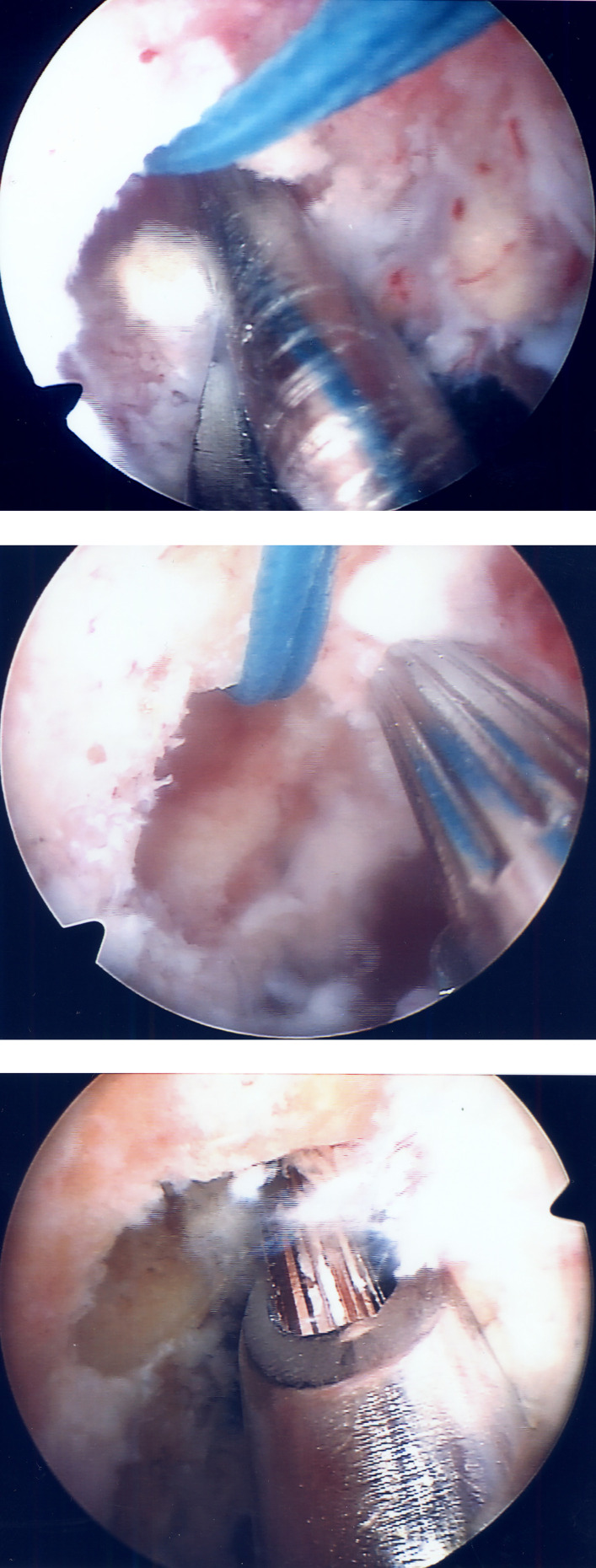
(a) Twice assessed per patient if the transtibial aiming K-wire a) aligned with the femoral tunnel area, within its perimeter (Group TI). (b) If it reached the area outside the femoral tunnel perimeter (Group TO). (c) Ιf it reached the femoral tunnel perimeter (Group TP).

The tibial tunnel was created using a tibial aiming device set at 50° angle (Figure 2). The guide sleeve was positioned approximately 1 cm above the pes anserinus and 1.5–2 cm medial to the tibial tubercle, forming a tibial tunnel angle of 65° in both coronal and sagittal planes [7] (Figure 3). A K-wire was positioned posterior to the center of the ACL footprint, documented fluoroscopically, and checked for impingement with a special impingement-checking instrument [7, 15]. The tibial tunnel was then drilled using a bit matching the graft diameter. For transtibial aiming, a 4-mm offset femoral guide was introduced through the tibial tunnel, targeting the center of the joint entrance of the femoral tunnel. This step was documented with videos and photographs (Figures 4a–4c).

#### Postoperative Stage

Two independent observers, both experienced orthopedic surgeons specializing in knee surgery, analyzed the postoperative data. They received anonymized patient files containing digital photographs and radiological images. Observers were instructed to measure the angles of the tibial tunnel in anteroposterior and sagittal views and to evaluate the position of the transtibial K-wire relative to the femoral tunnel. The K-wire position was categorized as being within the femoral tunnel perimeter (Group TI), at the perimeter (Group TP), or outside the perimeter (Group TO) [16]. These results were correlated with tibial tunnel direction (angle) on coronal and lateral X-rays, as well as the radius of the femoral tunnel recorded in the operative report.

### Statistical Analysis

Continuous variables are represented by the number of patients (*N*), mean value (mean), standard deviation (SD), and standard error of the mean (SEM). Categorical variables are presented using frequencies (*n*) and percentages (%).

Interobserver and intraobserver analyses for continuous variables were performed using the paired samples *t*-test. Interobserver analysis for categorical variables was conducted using the Kappa value of agreement, while intraobserver analysis for categorical variables was evaluated using McNemar’s test. Comparison between the two techniques was assessed using Fisher’s exact test. All tests are two-sided with a 95% significance level. Statistical analysis was performed using the statistical package SPSS version 10.00 (Statistical Package for the Social Sciences).

## Results

The accuracy of intraobserver and interobserver measurements was a primary focus of the study. The first observer demonstrated consistent intraobserver reliability, with minimal deviation between repeated measurements, resulting in a *p*-value of 0.694. The second observer exhibited greater variability between their first and second measurements, with the second measurement showing more deviation compared to the first observer (*p* = 0.05). Interobserver reliability showed that the second observer had more variability during their second measurement compared to the first observer (*p* = 0.007).

Radiological evaluation of the tibial tunnel positioning showed an average accuracy of 41.21%, with a range of 34–46.6% [17]. The femoral tunnel diameters varied among patients, with 21 cases having a diameter of 7 mm, 11 cases 7.5 mm, 7 cases 8 mm, 3 cases 8.5 mm, and 1 case 9 mm. Observer agreement was analyzed systematically. The first observer achieved 88.4% agreement between their measurements, with a Kappa index of 0.785, indicating consistent reliability. The second observer achieved an agreement rate of 95.4%, with a Kappa index of 0.906, reflecting high reliability. Interobserver reliability was also high, with agreement rates of 90.7% for the first evaluation and 95.4% for the second evaluation. Discrepancies were noted in five cases, which were excluded from this part of the analysis due to a lack of consensus.

The evaluation of transtibial aiming accuracy showed variability in placement outcomes (*p* < 0.0005). Among the 38 evaluated cases, 11 cases (29%) showed the targeting within the femoral tunnel perimeter. In 24 cases (63%), the targeting occurred outside the femoral tunnel perimeter. In three cases (8%), the targeting was at the perimeter of the femoral tunnel. These findings align with the final quantitative evaluations described in the methodology [17].

## Discussion

The goal of this clinical study was to evaluate the accuracy of femoral tunnel placement in single-bundle ACL reconstruction via two different techniques: the transtibial approach and the anteromedial portal approach. Our findings indicate that the transtibial technique, when aimed at the femoral tunnel deviates from the target femoral footprint compared to the anteromedial portal approach, which demonstrated greater precision. However, no significant correlation was found between the tibial tunnel inclination angles or tunnel diameter and the accuracy of femoral tunnel placement [6, 18].

The primary question of this study, regarding the accuracy of femoral tunnel positioning, revealed that the deviation between the entry point of the transtibial femoral guide and the center of the femoral tunnel entrance was approximately 4.50 mm. This result emphasizes the challenges inherent in the transtibial approach, particularly in achieving accurate femoral tunnel placement. Despite this, the findings suggest that the transtibial technique can still be considered viable, although it is less accurate than the anteromedial portal technique, especially when aiming for precise anatomical tunnel placement [6, 19, 20].

In terms of the secondary question, we found that tibial tunnel angles did not significantly influence the accuracy of the femoral tunnel placement. This suggests that the success of femoral tunnel positioning may not be as dependent on tibial tunnel alignment as previously thought. Further studies are required to explore the underlying mechanisms for this lack of correlation [21–23].

As with any study, certain limitations should be acknowledged. One limitation is the small sample size of 43 patients, which may not fully represent the diversity of cases encountered in broader clinical practice. Additionally, the study focused on a single surgeon’s technique, potentially limiting the generalizability of the results to other practitioners with different levels of experience. The use of fluoroscopy for radiological confirmation of tunnel positioning, while helpful, does not fully eliminate the risk of human error in the measurement process. Lastly, as the study was observational, we were unable to establish a direct causal relationship between the technique used and clinical outcomes, such as postoperative knee stability or functional recovery [24].

The authors’ experience in ACL reconstruction, particularly in refining techniques for tunnel placement, played a key role in the success of this study. With extensive practice, the surgeons were able to navigate the complexities of both methods, understanding their limitations and advantages. This experience was integral in achieving reliable results and providing insight into which technique may be most suitable depending on the surgical context and knee anatomy.

In conclusion, while both techniques are viable, the anteromedial portal approach provides greater accuracy in femoral tunnel placement in single-bundle ACL reconstruction. The transtibial approach, though still commonly used, presents more challenges and should be considered with caution in cases requiring high precision. Future studies with larger, more diverse patient populations and long-term follow-up are needed to confirm these findings and further refine ACL reconstruction techniques [7, 8, 21, 25].

## Data Availability

Data available on request due to privacy/ethical restrictions.

## Memorandum

Dr. H.H. Paessler organized and contributed significantly to this medical research, sharing his extensive expertise in orthopedic surgery, particularly in ACL reconstruction. His innovative approaches and dedication to advancing knee surgery have left a lasting impact on the field. Dr. Paessler’s contributions continue to inspire orthopedic surgeons and researchers worldwide, and his legacy remains a cornerstone in the advancement of orthopedic practice.
